# The Influence of Chemical Structure and the Presence of Ascorbic Acid on Anthocyanins Stability and Spectral Properties in Purified Model Systems

**DOI:** 10.3390/foods8060207

**Published:** 2019-06-12

**Authors:** Rachel Levy, Zoya Okun, Avi Shpigelman

**Affiliations:** Faculty of Biotechnology & Food Engineering, Russell Berrie Nanotechnology Institute, Technion, Israel Institute of Technology, Haifa 3200003, Israel; rachel447@campus.technion.ac.il (R.L.); zoya@bfe.technion.ac.il (Z.O.)

**Keywords:** anthocyanins, ascorbic acid, UV-Vis, HPLC-MS, kinetics, shelf life

## Abstract

The loss of color pigment is an important quality factor of food products. This work aimed to systematically study, in purified model systems, the influence of anthocyanins’ structure (by increasing the size of the conjugated sugar) and the presence of ascorbic acid on their stability and spectral properties during storage at two pH levels relevant to medium and high acid foods (6.5 and 4.5, respectively). Anthocyanins (cyanidin (Cy), cyanidin 3-O-β-glucoside (Cy3G) and cyanidin 3-O-β-rutinoside (Cy3R)) displayed first-order degradation rates, presenting higher stability in acidic medium and enhanced stability with increasing size of conjugated sugar. The addition of ascorbic acid resulted in significantly enhanced degradation. Changes in ultra violet visible (UV-VIS) spectral properties presented a decrease in typical color intensity and pointed towards formation of degradation products. Identification and kinetics of formation for cyanidin degradation products were obtained by high performance liquid chromatography system-mass spectrometry (HPLC-MS).

## 1. Introduction

Color is an important sensory characteristic that consumers rely on when making food choices, and even the slightest changes due to processing or during shelf life can deter a potential buyer and alter the consumer’s perception of product quality. The loss of color pigment is an important shelf life factor for many food products, especially for those undergoing thermal treatment [1]. Anthocyanins are a large group of natural water-soluble pigments that are responsible for red, blue and purple colors in many fruits, vegetables, flowers, leaves, stems and roots [2,3], and their deterioration is an important factor in loss of color in many food products [1]. The bright colors of the pigments are due to the conjugated double bonds that are responsible for the absorption of visible light [4], and the pH dependent equilibrium allows their utilization as natural food colorants [3,5]. These pigments usually occur in the glycosylated polyhydroxy and polymethoxy form of 2-phenylbenzopyrylium salts (flavylium ion skeleton). Beyond their effect on color, the increasing interest in the research of these molecules stems from their reported various health promoting properties such as anti-inflammatory [6], anti-cancer [7] anti-cardiovascular [8] and other bioactivities [8,9,10,11]. As members of the polyphenol group, anthocyanins and anthocyanidins (the aglycone form of anthocyanins) possess antioxidant properties [8,9], the conjugated sugar moiety of the anthocyanins is known to reduce the radical scavenging activity as compared to aglycone [8,12]. It was suggested that attached sugar unit reduces the ability of the anthocyanin radical to delocalize electrons [8].

While anthocyanins are widely occurring in food products, they tend to be unstable during processing and storage and to be degraded and/or decolorized. Some studies explore the chemistry and stability of anthocyanins, mostly in fruit and vegetables and their processed products, but also in some model systems [9,12,13,14,15,16]. The stability is reported to be affected by pH, temperature, chemical structure of the pigment, concentration, solvents, oxygen, light, enzymes, etc. [2,3,9,17,18]. During processing and storage, anthocyanins degradation increases with the rise in temperature [13,19]. At room temperature, color is reported to be stable only in acidic media. In alkaline media, cleavage of the pyrylium ring takes place and decrease in color intensity is noticed [20]. It might be assumed that thermal degradation of anthocyanins begins in the opening of the heterocycle and the formation of the chalcone form [21,22]. Heating shifts the equilibrium towards the chalcone and the reversion of chalcone to flavylium is slow to impossible [22]. Thermal degradation of anthocyanins follows first-order reaction kinetics [23,24,25] and can be reduced by decreasing the pH [1,25]. Another suggested mechanism excludes the formation of chalcone glycoside form, and by combination of heat and pH levels (2–4) hydrolysis of the glycosidic bond occurs, followed by conversion of the aglycone to chalcone and then to degradation products [22]. At aqueous solution at pH 2–4, temperature elevation leads to the hydrolysis of glycosidic bond, resulting in the loss of sugar moieties of the anthocyanins, leading to further loss of color as the anthocyanidins are much less stable than anthocyanins [20]. The presence of the sugar group is responsible for increased water solubility and stability (compared to aglycone) [4,26], with the number of sugar rings also suggested to influence the stability [22,27,28].

Many food products, especially juices, are fortified with ascorbic acid (AA), a natural antioxidant, to protect against oxidation and to increase the nutritional value [29,30]. AA might have several negative influences on anthocyanins’ stability. In the presence of oxygen, AA can accelerate the degradation of anthocyanins and enhance the formation of polymer pigment, which results in anthocyanins pigment bleaching [29,30,31]. The exact mechanism is still controversial and addition of AA to anthocyanins results in increase of the degradation rate of both molecules. The postulated mechanisms are either direct condensation of AA with anthocyanins or formation of hydrogen peroxide and oxidative cleavage of the pyrylium ring by peroxide [26,29,31]. In previous works, the focus of anthocyanins stability in the presence of AA is explored in food matrixes [32,33] or with a focus on specific molecules [29,31].

The aim of this study was to explore, systematically, in purified model systems, the influence of anthocyanins’ structure (by increasing the size of the conjugated sugar), pH (6.5 and 4.5) and the presence of ascorbic acid on their stability and spectral properties during simulated shelf life. The degradation was tested in purified model systems to avoid possible interferences from food matrix. In addition, we aimed to better understand the kinetics of the formation of degradation products for the most sensitive of the tested anthocyanins—cyanidin. To our knowledge, no other work examines the stability of series of three molecules that differ only by the presence and type of sugar moiety, by high performance liquid chromatography system-mass spectrometry (HPLC-MS) analysis and quantification, monitoring of the spectral properties and identification of the degradation products in purified system.

## 2. Materials and Methods

### 2.1. Materials

Cyanidin chloride (Cy) was purchased from Tokiwa phytochemical Co., LTD (Tokyo, Japan) (CAS number: 528-58-5). Cyanidin 3-O-β-glucopyranoside (CAS number: 7084-24-4) and Cyanidin 3-O-(6′′-O-α-rhamnopyranosyl-β-glucopyranoside) (CAS number: 18719-76-1) were purchased from Polyphenols AS (Sandnes, Norway). l-(+)-Ascorbic acid 99+%, was purchased from Alfa Aesar (Heysham, England) (CAS number: 50-81-7). 2,4,6-trihydroxybenzaldehyde (CAS number: 487-70-7) and 2,4,6-trihydroxybenzoic acid (CAS number: 71989-93-0) were purchased from Sigma-Aldrich (Saint Louis, MO, USA). 3,4-dihydroxybenzoic acid was purchased from Fluka Chemika (Buchs, Switzerland) (CAS number: 99-50-3).

### 2.2. Methods

#### 2.2.1. Buffered and Stock Solutions

Stock solutions of Cy3G, Cy3R, 2,4,6-trihydroxybenzaldehyde, 2,4,6-trihydroxybenzoic acid and 3,4-dihydroxybenzoic acid (2.5 mM) were prepared in pure methanol. Cy stock solution (2.5 mM) was prepared in methanol and 1% (*v*/*v*) formic acid. All stock solutions were covered with aluminum foil and stored at −40 °C. Acetate buffered solution (20 mM, pH = 4.5) was prepared using acetic acid and sodium acetate. Phosphate buffered solutions (20 mM, pH = 1.5 and pH = 2.5) were prepared using phosphoric acid. Phosphate buffered solution (20 mM, pH = 6.5) was prepared using sodium phosphate, monobasic and dibasic.

#### 2.2.2. Preparation of Anthocyanins Model Solutions

The stability of Cy, Cy3G and Cy3R was conducted by diluting the Cy, Cy3G, and Cy3R stock solutions in phosphate buffered solution pH = 6.5 and acetate buffered solution pH = 4.5 for final concentration of 0.1 mM (4% methanol). For exploring the influence of AA, similar solutions were prepared with an addition of AA (200 mg/L) the pH of the samples was adjusted correspondingly. Appropriate controls of AA without anthocyanins and pure buffered solutions were prepared. The samples were vortexed and stored at least in duplicate in the dark at 15, 23 and 37 °C. Immediately after preparation (*t* = 0) and after 6 h, 27 h, 32 h, 72 h and 146 h, the samples were diluted with acidic buffered solutions (pH = 1.5 for Cy and pH = 2 for Cy3G and Cy3R) for final concentration of 0.05 mM (2% methanol) to get the maximum stability of the anthocyanins for HPLC-MS analysis. The samples when then frozen and stored at −40 °C until analysis.

#### 2.2.3. Quantification of the Model Solutions Degradation and Degradation Products

The acidified samples were filtered by a 0.45 µm PVDF syringe filters (Merck Millipore LTD., Carrigtwohill, Ireland) and injected to an Agilent 1260 high performance liquid chromatography system with mass spectrometry (HPLC-MS) 6120 Quadrupole (Santa Clara, CA, USA) equipped with InfinityLab Poroshell 120 EC-C18 column (2.1 × 150 mm, 1.9 micron) protected by guard column (Agilent, Santa Clara, CA, USA). The injection volume was 10 µL. The flow rate was 0.19 mL/min, column temperature was set to 30 °C and the sampler temperature was set to 4 °C. The mobile phase included: an aqueous solution of 1% formic acid (A) and an aqueous solution of 50% 1% formic acid, 25% methanol and 25% acetonitrile (B). A gradient of 25% B for 0–15 min, 100% B for 15–17 min, 25% B for 17–22 min and 25% B for 22–23 min was used. The chromatograms were acquired at 270 nm, to detect not only the anthocyanins and at 516 nm for anthocyanins quantification. All analysis in the HPLC-MS were carried out in full scan mode from 150 to 870 *m/z* API-ES source in negative and positive mode. The capillary: negative 3500 V, positive 4000 V, nebulizer gas (N2) 60 psi, dry gas (N2) 13 (1/min), dry temperature 350 °C. Validation of the Cy, Cy3G, and Cy3R peaks was performed by negative (286, 448, and 594) or positive (288, 450, and 596) selected ion measurement (SIM), respectively. Relative concentration of the remaining anthocyanins for each sample were calculated as the peak area of experimental samples divided by the average area of the same sample at *t* = 0 min at 516 nm. For the quantification of Cy degradation products, relative concentration of degradation products was calculated at 270 nm as the average peak area of experimental samples divided by the maximum average area of the samples over the experimental time.

#### 2.2.4. Qualitative Spectroscopic Analysis of Model Solutions

For qualitative study of the color stability, all samples of model solution were loaded on a 96-well quartz microplate. The appropriate controls containing AA in buffered solutions without anthocyanins and pure buffered solutions were prepared and also loaded on the microplate. The samples’ ultra violet visible (UV-VIS) absorbance was recorded as a function of time at the range of 250–600 nm using a Synergy H1 hybrid multi-mode reader (Biotek, Winooski, VT, USA) connected to a computer. The plate was stored in the dark at all the examined temperatures.

#### 2.2.5. Calculation of Reaction Rate Constants

The first-order reaction rate constants (k) were calculated by nonlinear curve fitting using Origin 2018 (OriginLab, Northampton, MA, USA).

#### 2.2.6. Statistical Analysis

The significance of the influence of chemical structure on stability was calculated using the nonlinear model to compare multiple databases by Origin 2018 (OriginLab), assessing if two datasets were significantly different from each other by an F-test (*p* < 0.05).

## 3. Results and Discussion

### 3.1. Degradation of Cyanidin

#### 3.1.1. Stability of Stock Solution

One of our goals was to characterize the degradation of cyanidin by spectral changes and the formation of degradation products. Therefore, we first aimed to find the most stable conditions for preparation of Cy stock solution. From the tested solvents and storage conditions, only storage in methanol with 1% formic acid at −40 °C allowed conservation of more than 95% of Cy after 30 days. In all other tested stock solutions (pure methanol or methanol:water (20:80 and 50:50) with 1% formic acid, buffers at pH 2–7 stored at −20 °C and −40 °C), degradation of Cy was detected by HPLC-UV resulting in formation of new peaks.

#### 3.1.2. Stability of Cyanidin and the Formation of Degradation Products

Anthocyanins are more stable in acidic solutions than in alkaline or neutral media [22]. In solution media, four main equilibria forms are known: flavylium cation, quinoidal base, carbinol pseudobase and chalcone (*cis* or *trans*). In acidic solutions up to pH 3, the main form is flavylium cation (red color). By raising the pH above 4, the color pigment and concentration of the flavylium cation decrease and the color can turn blue due to quinoidal base or even colorless or yellowish pigment due to chalcone and pseudobase. The process might be reversible until the point where the pH value is too high and unstable ionic chalcone is formed. At this stage, the regeneration of color cannot be achieved. The chalcone was suggested to further degrade to 2,4,6-trihydroxybenzaldehyde and phenolic compound or coumarin [3,34]. In many food products containing anthocyanin, the pH range is 2–4 resulting in the flavylium cation as the main specie [34,35].

**Figure 1:**
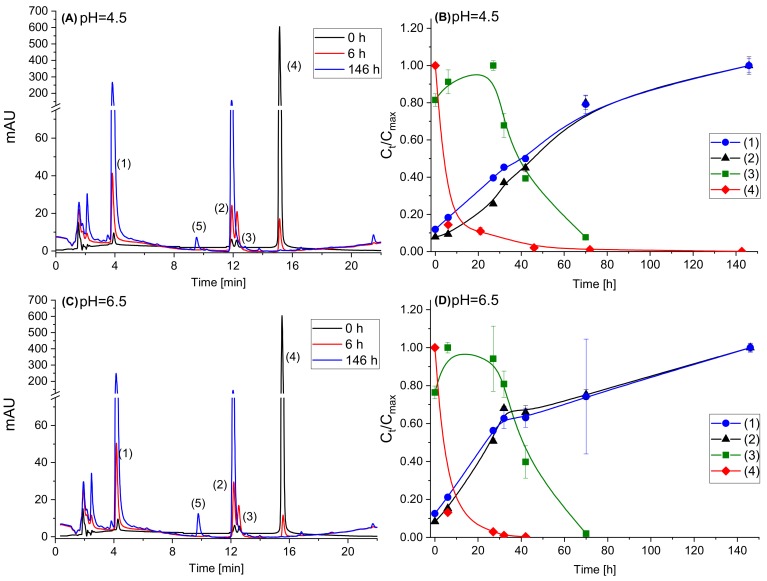
Stability of Cy and the formation of degradation products in buffered solutions stored at 37 °C: (**A**) a typical HPLC chromatogram at 270 nm of Cy solution (pH = 4.5) after 0, 6 and 146 h; (**B**) relative concentration (Ct/Cmax) of Cy and Cy degradation products (pH = 4.5); (**C**) a typical HPLC chromatogram at 270 nm of Cy solution (pH = 6.5) after 0, 6 and 146 h; and (**D**) relative concentration (Ct/Cmax) of Cy and Cy degradation products (pH = 6.5). Compounds identification: (1) 3,4-dihydroxybenzoic acid (Rt = 3.8); (2) 2,4,6-trihydroxybenzaldehyde (Rt = 11.9); (3) chalcone (Rt = 12.2); (4) Cy (Rt = 15.9); and (5) unidentified degradation product (Rt = 9.5). Quantification was made by HPLC-UV absorbance of the peak at 270 nm. Error bars represent standard error (*n* = 2). In some cases, they are smaller than the symbols. The lines in (**B**,**D**) are to guide the readers’ eye.

**Figure 2:**
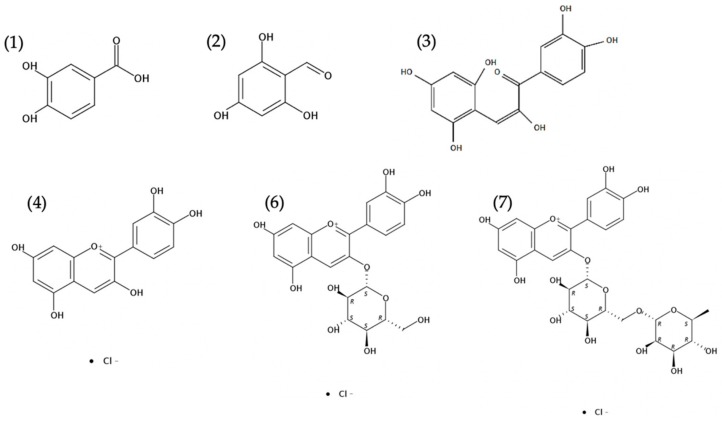
Chemical structure of anthocyanins and suggested degradation products: (**1**) 3,4-dihydroxybenzoic acid; (**2**) 2,4,6-trihydroxybenzaldehyde; (**3**) chalcone; (**4**) Cyanidin (Cy); (**6**) Cyanidin 3-O-β-glucoside (Cy3G); and (**7**) Cyanidin 3-O-β-rutinoside (Cy3R)). Structures were obtained using SciFinder^®^ application.

The stability of Cy during 146 h at 37 °C and two pH values, 4.5 and 6.5, relevant to mild and low acid foods, was monitored by HPLC-UV-MS (Figure 1). The presented chromatograms in Figure 1A,C clearly shows the degradation of Cy (Rt = 15.9, peak number (4)) over time at both pH values. No significant difference between the degradation of Cy in pH = 4.5 compared to pH = 6.5 was noticed, possibly due to the quick degradation at both pH conditions. The chromatograms also clearly present, for the first time in non-highly acidic conditions, the kinetics of formation of the degradation products. Reported works identify possible degradation products of Cy as chalcone (cis or trans), 3,4-dihydroxybenzoic acid, 2,4,6-trihydroxybenzaldehyde and 2,4,6-trihydroxybenzoic acid (Figure 2) yet in pH values not relevant for food products [13,19]. In this study, three main degradation products were identified and monitored over time (Figure 1B,D): 3,4-dihydroxybenzoic acid (Rt = 3.8, peak number (1)), 2,4,6-trihydroxybenzaldehyde (Rt = 11.9, peak number (2)) and chalcone (cis or trans, Rt = 12.2, peak number (3)). The peaks of 3,4-dihydroxybenzoic acid and 2,4,6-trihydroxybenzaldehyde were compared to standards and identified by retention time (Rt), absorption spectra and mass spectral analysis. In both distinct pH levels, the same formed degradation products were observed and the general trend of their formation and further degradation was similar. At pH = 4.5 and pH = 6.5, the chalcones appeared up to 32 h and 6 h respectively, and then decreased with time. These results support previous suggested mechanism [13] showing that chalcones are being consumed in subsequent degradation process and are intermediate deterioration products. At the same time, the amount of the other two products continuously increased with time. Additional suggested secondary degradation product, 2,4,6-trihydroxybenzoic acid [13,19], was not identified in any of the systems during the examined time. After 146 h, a new peak (5) (Rt = 9.5) appeared at both pH values only at the chromatograms acquired at 270 nm with fragmentation of 289 (as the major mass in the mass spectrum), but it was not identified.

**Figure 3:**
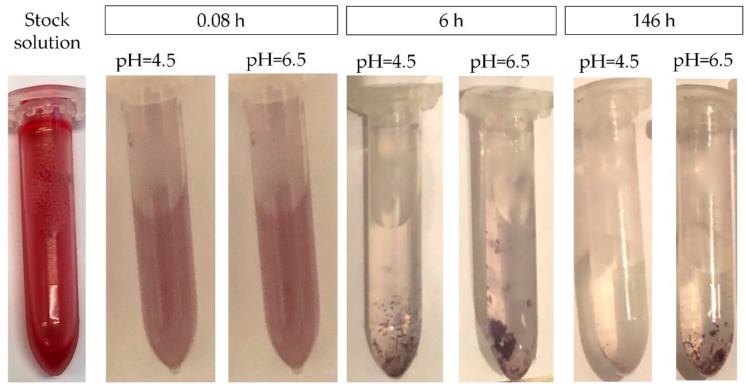
Visual color of Cy stock solution and Cy in buffered solution pH = 4.5 and pH = 6.5, stored at 37 °C after 5 min, 6 h, 42 h and 143 h.

The visual appearance of the Cy stock (2.5 mM) and Cy sample (0.1 mM) solutions over time are presented in Figure 3. Cy concentrations were below the reported maximal solubility of Cy (0.17 mM) which is the least soluble from the three studied molecules [36]. The color of Cy immediately changed from red into blue-purple at both pH values. After 6 h, the formation of insoluble purple sediment was observed at all studied temperatures (15 °C, 23 °C, and 37 °C). While chemical instability of anthocyanins is well documented, to the best of our knowledge, the formation of physical instability (sediment) during shelf-life/storage is not reported before. We suggest that the observed sediment is, at least partially, the outcome of chemical instability resulting in the formation of insoluble degradation products that would not be detected in the chromatograms in Figure 1A,C, although we cannot exclude involvement of Cy molecules themselves. The identification of the purple residues, the kinetics and factors affecting their formation should be further studied as the sediment may have a major impact on both sensorial and nutritional properties.

### 3.2. The Influence of Chemical Structure of Anthocyanins on Stability and Spectral Properties

#### 3.2.1. Stability of Stock Solutions and Buffered Solutions

Cy3G and Cy3R stock solutions were prepared in pure methanol and stored at −40 °C allowing the conservation of more than 98% of the original compounds after 30 days. In buffered solutions that were also tested for stock formation, the lowest degradation of these molecules was observed in phosphate buffered solution pH = 2.5.

#### 3.2.2. The influence of the Sugar Moiety Size

**Figure 4:**
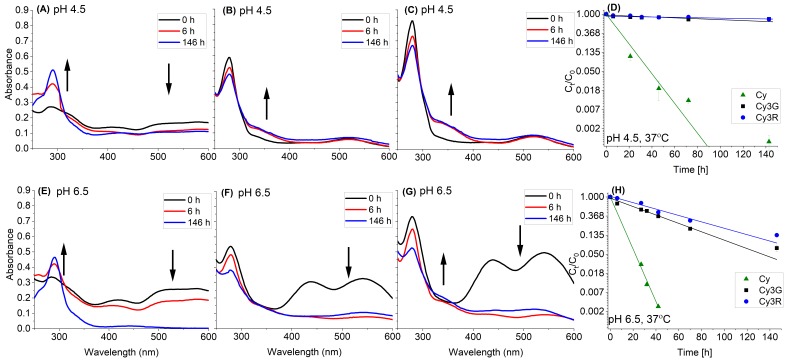
Stability (by HPLC) and changes in the absorbance spectrum of Cy, Cy3G and Cy3R stored at 37 °C: (**A**) average absorbance spectrum of Cy (pH = 4.5); (**B**) average absorbance spectrum of Cy3G (pH = 4.5); (**C**) average absorbance spectrum of Cy3R (pH = 4.5); (**D**) relative concentration (compared to *t* = 0) of Cy, Cy3G and Cy3R over time (pH = 4.5); (**E**) average absorbance spectrum of Cy (pH = 6.5); (**F**) average absorbance spectrum of Cy3G (pH = 6.5); (**G**) average absorbance spectrum of Cy3R (pH = 6.5); and (**H**) relative concentration (compared to *t* = 0) Cy, Cy3G and Cy3R over time (pH = 6.5). Quantification was made by HPLC-VIS absorbance of the peak at 516 nm and presented as percentage of the peak area divided by the initial peak area. (**D**,**H**) Error bars represent standard error (*n* = 2); in some cases, they are smaller than the symbols. The linear line represents fit to first-order degradation kinetics.

To understand the influence of the size of the conjugated sugar moiety, the stability of Cy, Cy3G and Cy3R was monitored at different pH conditions (4.5 and 6.5) and temperatures (37 °C, 23 °C, and 15 °C). Figure 4 presents stability results as the relative concentration of the peak area divided by the initial peak area at 516 nm for the compounds stored at 37 °C. In addition, the changes in the spectral properties were monitored by collecting UV-VIS absorbance spectra over time.

Cy degradation was immediate; Cy3G and Cy3R presented significantly higher stability than Cy at both pH values (*p* < 0.05) (Figure 4D,H). These results support the hypothesis that the sugar moiety stabilizes the molecules [20,22] and that the decrease in absorbance might be used as indicator for the formation of degradation products [13,26,31]. While the decrease in absorbance is often used as a simple method for quantification of anthocyanins degradation [14,28], at the higher pH, the correlation between concentrations quantified by HPLC and the decrease in the typical VIS absorbance above 500 nm (measured by spectrophotometer) seems less clear (Figure 4G,H), therefore should be verified and treated with caution. This could stem from the various intermediate reversible products that are likely to appear at higher pH levels. Beside the decrease in color intensity in the visible range, increase in the absorbance was observed in the UV range for all samples at pH 4.5 and in Cy solutions in pH 6.5 (Figure 4A–C,E). This is in good agreement with the degradation products detected in the HPLC-UV elution profile of all samples at 270 nm. When comparing the size of the sugar moiety, a significant difference (in the value of the slope, k) between the degradation of Cy3G and Cy3R in the two pH values (*p* < 0.05) was detected, although the effect was larger at pH 6.5 (Figure 4D,H). This result further presents that the disaccharide moiety stabilizes the anthocyanin more than the monosaccharide. In addition, at alkaline solutions, the decrease in stability of Cy3G and Cy3R, was faster (*p* < 0.05), as expected [22]. In contrast, there was no significant difference in the stability of Cy depending on the pH value, yet further work verifying if this is the outcome of extremely fast degradation at both pH levels or a mechanism that is not as pH depended (in these pH range) as in Cy3G and Cy3R is needed.

The complete anthocyanin thermal degradation mechanism is still unclear [21,27,37]. As mentioned, it was suggested before that thermal degradation of anthocyanins in aerobic conditions and buffer solution pH = 2–4 starts with the formation of chalcone glycoside followed by hydrolysis of the sugar moieties [21]. Another proposed mechanism suggested hydrolysis of the sugar moieties and then formation of chalcone [29]. However, after the formation of the chalcone, the degradation products of anthocyanins should eventually be the same as those of the aglycone [21]. In our HPLC-MS analysis of Cy3G and Cy3R solutions at both pH values (data not shown), a decrease in the peak areas of Cy3G and Cy3R was clearly observed, followed by the appearance of new peaks detectable at 270 nm. However, degradation products of Cy3G and Cy3R have not yet been fully characterized.

### 3.3. The Influence of the AA Addition on the Stability of Anthocyanins

**Figure 5:**
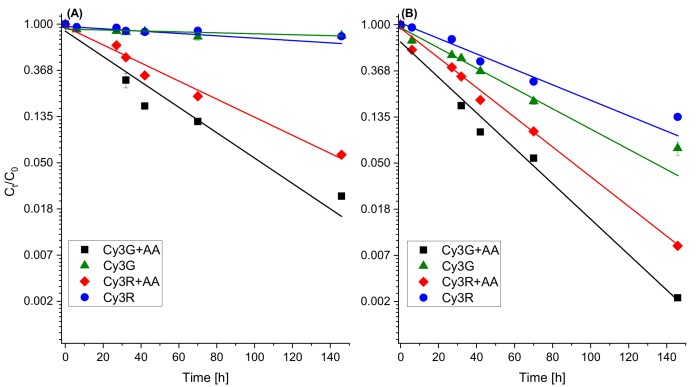
Stability of Cy3G and Cy3R in buffered solutions (pH 4.5 (**A**); and pH 6.5 (**B**)) over time with and without AA, stored at 37 °C. Quantification was made by HPLC absorbance of the peak at 516 nm and presented as relative concentration of the peak area divided by the initial peak area. Error bars represent standard error (*n* = 2). In most cases, they are smaller than the symbols.

To better understand the influence of AA addition to anthocyanins, the stability of Cy3G and Cy3R was measured during time at different pH conditions. The fact that this work focuses only on two isolated molecules without the presence of food matrix allows a better understanding of the effects of AA on anthocyanin stability. The concentration of AA used in the model system, 200 mg/L, was similar to that found in fruit juices [38]. Stability results are presented as relative (compared to *t* = 0) concentrations quantified as peak area at 516 nm at the two pH values (Figure 5).

It is known that AA in the presence of oxygen accelerates the decomposition of several anthocyanins and leads to bleaching of anthocyanin pigments [29,30,31,39]. Our results show that, after 6 h, there was a drastic decrease in the peak area of AA (by HPLC, areas are not shown) at 270 nm in the solution with anthocyanins compared to the control solution (only AA). The degradation mechanism of anthocyanins in the presence of AA is controversial. The postulated mechanisms are either direct condensation of AA with anthocyanins [40] or by free radical mechanism (formation of hydrogen peroxide and oxidative cleavage of the pyrylium ring by this peroxide) [26,31]. In all model systems, with and without the addition of AA, decrease in the peak areas of Cy3G and Cy3R were followed by appearance of new peaks in the HPLC chromatogram. Difference in the HPLC chromatogram in all systems containing AA compared to the corresponding systems without AA were observed, yet no clear identifiable condensation products between AA with Cy3G and Cy3R were detected.

The stability results of Cy3G and Cy3R indicate that there is a significant increase in the degradation in the presence of AA (*p* < 0.05), with residual concentrations after 146 h of less than 6% for the samples containing AA compared to 77% when AA was not added at pH 4.5 (Figure 4). Figure 4 also reveals that pH has an influence on the degradation rate, as the solution is more acidic the anthocyanin is more stable with and without added AA (*p* < 0.05). The size of the conjugated sugar moiety also has an influence; for both pH values in the presence of AA, Cy3R was more stable than Cy3G, and even more clearly observed at pH 4.5 compared to the system without AA. As the pH value might influence the activity of AA and therefore the degradation of anthocyanins, this point needs further structured in-depth research.

Anthocyanins deterioration is known to follow first-order degradation rate [23,24,25]. To compare and understand the influence of chemical structure and the presence of AA on the stability of anthocyanins, degradation rate constants (k) were calculated for each explored molecule, with and without the presence of AA at different buffers and temperatures (15 °C, 23 °C and 37 °C). The data are summarized in Table 1. The results present a significant (*p* < 0.05) increase in k values as the storage temperature increased for most systems, with practically no differences between the storage conditions for the lowest studied temperature. The occasionally lacking hypothesized statistically significant differences at the lower temperatures likely originate from the relative stability at the lower temperatures, making statistical verification of differences harder. However, at longer storage times, the presented effects of AA, pH and size of the conjugated sugar are expected to be identifiable also at lower temperatures. The presence of AA significantly accelerated the degradation of anthocyanins stored at 37 °C (p < 0.05). At pH = 4.5, without added AA, both Cy3R and Cy3G have no significant differences between the k values in the three explored temperatures, likely due to the relatively high stability at this pH. However, at pH = 6.5, there are significant differences (*p* < 0.05) between the k values at the different temperatures.

## 4. Conclusions

We present a systematic stability study, based on both spectral and HPLC methods, focusing on the effect of cyanidin derivatives structure on the kinetics and outcome of non-enzymatic deterioration. The materials were examined during simulated storage for up to six days in different pH and temperature conditions, with and without AA presence, and the results were analyzed by HPLC-MS and spectral studies. The rapid and high anthocyanins degradation rates, especially when stored without cooling, indicate that it is highly important to identify the mechanisms of non-enzymatic deterioration in the presence of common food formulations, for such health promoting pigments. The anthocyanin chemical structure has an influence on stability and on color, presenting complete lack of stability of the anthocyanidin, resulting in both soluble and insoluble molecules. Further studies are required to fully uncover the mechanism responsible for the higher stability of anthocyanins conjugated to a di-saccharide compared to a mono-saccharide moiety. A decrease in the typical anthocyanin color was observed for all anthocyanins depending on the structure and pH level, yet at the higher pH it was not fully correlated to the original compound degradation as measured by HPLC. Degradation products of Cy were identified and quantified over time by HPLC-MS presenting a complex multi-step degradation reaction. Degradation rate constants were calculated for Cy3G and Cy3R in buffer solutions with and without the addition of AA. When aiming for maximal stabilization of anthocyanins during storage, our results clearly show that the use of AA should be avoided as AA led to a drastic reduction in Cy3G and Cy3R content in both studied pH levels that are representative of mild and low acid products. Complete degradation products profile in buffer solution with and without AA has not been achieved thus far. Future studies should focus not only on the quantification of degradation of the original compounds but also, as was attempted in this work, to identify and quantify the degradation products, in order to better understand the mechanisms allowing improved capabilities for maximizing shelf life. In addition, such research can help in understanding the possible nutritional outcome of anthocyanins deterioration as the degradation products can also have bioactivity.

## Figures and Tables

**Table 1:** Degradation rate constant, k (day^−1^), of Cy3G and Cy3R solutions in the presence of AA at pH = 4.5 and pH = 6.5 stored at 15 °C, 23 °C and 37 °C over time (*n* = 2).

T (°C)	Cy3G				Cy3R			
	4.5		6.5		4.5		6.5	
	AA (+)	AA (−)	AA (+)	AA (−)	AA (+)	AA (−)	AA (+)	AA (−)
15	0.036 ± 0.005 ^a^	0.057 ± 0.011 ^a,c^	0.054 ± 0.010 ^a,c^	0.135 ± 0.037 ^b,d^	0.043 ±0.006 ^a,c^	0.051 ± 0.020 ^a,b,c^	0.059 ± 0.003 ^c^	0.091 ± 0.041 ^d^
23	0.065 ± 0.004 ^a^	0.028 ± 0.008 ^b^	0.151 ± 0.014 ^c,e^	0.127 ± 0.015 ^c^	0.095 ± 0.009 ^d^	0.027 ± 0.011 ^b,e^	0.128 ± 0.012 ^e^	0.088 ± 0.008 ^a,e^
37	0.659 ± 0.073 ^a^	0.024 ± 0.005 ^b^	0.922 ± 0.065 ^c^	0.528 ± 0.040 ^d^	0.469 ± 0.023 ^d^	0.057 ± 0.006 ^b^	0.774 ± 0.008 ^e^	0.402 ± 0.042 ^f^

Different letters indicate significant changes at each of the studied temperature.
